# Kawasaki Disease: A Rare Case of a Non-pediatric Patient

**DOI:** 10.7759/cureus.74824

**Published:** 2024-11-30

**Authors:** Marta Ramos, Filipe Seguro Paula, Alexandre Carvalho, Marta Pinheiro, Armindo Ramos

**Affiliations:** 1 Intensive Care Unit, Hospital de Cascais, Lisboa, PRT; 2 Immune Response and Vascular Disease, NOVA Medical School, NOVA University of Lisbon, iNOVA4Health, Lisboa, PRT; 3 Systemic Immune-Mediated Diseases Unit (UDIMS), Hospital Fernando da Fonseca, Amadora, PRT

**Keywords:** adults, cardiology, intensive medicine, kawasaki disease, systemic vasculitis

## Abstract

Kawasaki disease (KD) is an inflammatory condition more common in children but rare in adults, where diagnosis can be challenging due to nonspecific symptoms. Early recognition is essential to prevent severe complications.

We present the case of a 26-year-old male with adult-onset KD who initially presented with vague symptoms, resulting in diagnostic delays.

The condition progressed to life-threatening cardiovascular complications, including coronary artery aneurysms, underscoring the critical need for prompt identification and management. Timely treatment with intravenous immunoglobulin and corticosteroids led to clinical improvement and prevention of further cardiac damage.

This case highlights the importance of heightened clinical awareness of KD in adults and the necessity of a proactive diagnostic approach. Future research should aim to refine diagnostic criteria for adult KD and explore strategies for improving early detection and long-term cardiovascular outcomes in this rare population.

## Introduction

Kawasaki disease (KD) is an acute systemic autoimmune vasculitis targeting small- and medium-size vessels with an unknown etiology. While rare, KD predominantly affects infants and young children and is decidedly uncommon in adults [1-8]. It is estimated that KD affects nine to 20 children per 100,000 who are under five years old in the United States. Due to its rarity in adults, there is no precise percentage estimate for this population [9].

The classical diagnosis of KD relies on the presence of fever lasting at least five days, along with the identification of at least four out of the five main findings [10]: bilateral non-exudative conjunctivitis, typical changes of the lips or oral mucosa (including erythema and cracking of the lips, "strawberry tongue" with erythema and prominent fungiform papillae, and/or erythema of the oral and pharyngeal mucosa), a maculopapular or erythema multiforme-like rash, changes in the peripheral extremities accompanied by subsequent periungual desquamation during the sub-acute phase, and cervical lymphadenopathy [1,3].

However, the diagnosis of KD remains challenging, especially in adults, as its clinical manifestations are often not present at the same time [1]. Moreover, the clinical features required for a KD diagnosis overlap with those of several infectious diseases [4]. Delayed or incorrect diagnoses are linked to a higher risk of coronary artery aneurysm formation, which can lead to irreversible heart failure resulting from myocardial ischemia due to coronary artery thrombosis and stenosis [1,5-7]. We present a rare and challenging case of adult-onset KD shock syndrome.

## Case presentation

A 26-year-old male with no prior medical history was admitted to the emergency department due to fever, headache, and throat pain. On admission, he was normotensive and normocardic, with no significant findings at observation. Initial blood tests (Table 1) showed a high C-reactive protein (CRP) of 21mg/dL (normal <0.5 mg/dL) and positive reactivity for IgG antibodies against Epstein-Barr virus (IgM negative), with no other worrisome findings. A neck computed tomography (CT) scan revealed an enlarged palatine tonsil, with no suspicion of local infectious complications. Viral tonsillitis was assumed, and he was discharged home on anti-inflammatory drug therapy. 

**Table 1:** Initial blood tests

Laboratory test	Result	Normal range
Platelets	157000/uL	150000-400000/uL
C-reactive protein (CPR)	21mg/dl	< 0.5mg/dl
IgG antibody Epstein-Barr	Positive	-
IgM antibody Epstein-Barr	Negative	-

**Figure 1:**
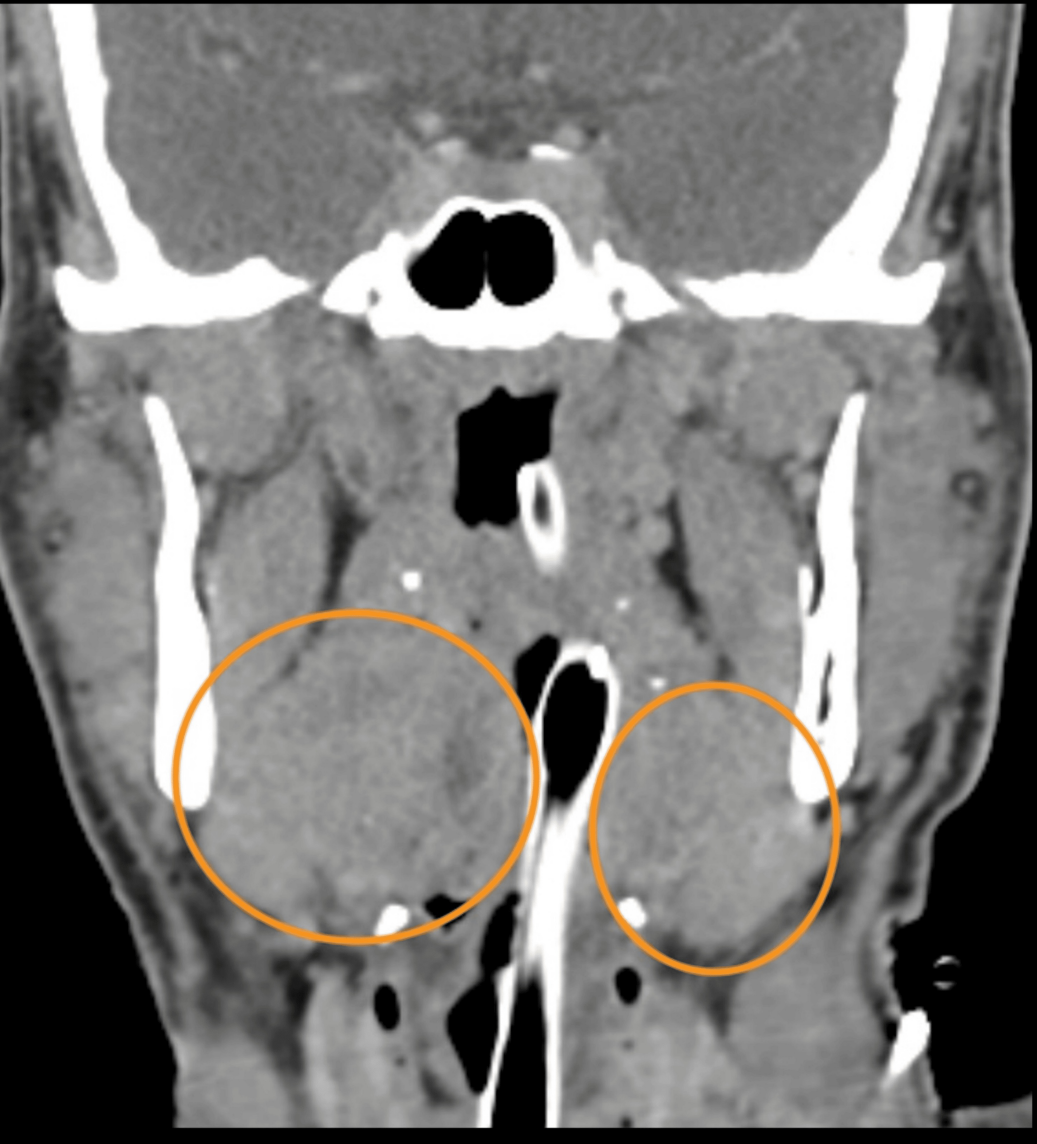
Neck CT scan showed an enlarged palatine tonsil (orange circle), without local infectious complications The tonsils are marked with orange circles. In the left circle: enlarged tonsil with no local complications. In the right circle: slight enlargement of the tonsil, with no local complications.

Three days later, he was re-admitted to the ED due to persistent symptoms. He was now lethargic, dehydrated, and hypotensive (blood pressure 60/30 mmHg) despite fluid resuscitation (four liters of crystalloid fluids). A mild cutaneous rash was noted. The blood tests are described in Table 2. A diagnosis of toxic shock secondary to tonsillitis was entertained; however, a repeat CT scan once again showed no signs of local tonsillar-related infectious complications, like abscesses (Figure 1), and other sources of infection were ruled out by microbiology screenings. The patient was admitted to the ICU with an initial diagnosis of sepsis/septic shock and multiorgan dysfunction, requiring vasopressor support, mechanical ventilation, and empiric antibiotic therapy while the diagnostic work-up was being pursued.

**Table 2:** Blood tests on admission to the emergency department

Laboratory test	Result	Normal range
Leucocytes	13800/uL	4000 -10000/uL
Lymphocytes	550/uL	1000 - 4000/uL
Neutrophils	81% (11178/uL)	1800 - 7000/uL
C-reactive protein (CPR)	27 mg/dl	< 0.5 mg/dl
Sedimentation velocity	87 mm/h	< 15 mm/h
Platelets	57000/uL	150000 - 400000/uL
Creatinine	2.7 mg/dl	0.7-1.3 mg/dl
AST	73 UI/L	15 – 37 UI/L
ALT	139 UI/L	46 – 116 UI/L
Total bilirubin	3.99 mg/dl	0.2 – 1.0 mg/dl
Triglycerides	887 mg/dl	50 - 199 mg/dL
Total cholesterol	165 mg/dl	< 200 mg/dl
Sodium	137 mmol/L	136 – 145 mmol/L.
HIV	Negative	-
Serum lactate	2.1 mmol/L	< 1.0 mmol/L

**Figure 2:**
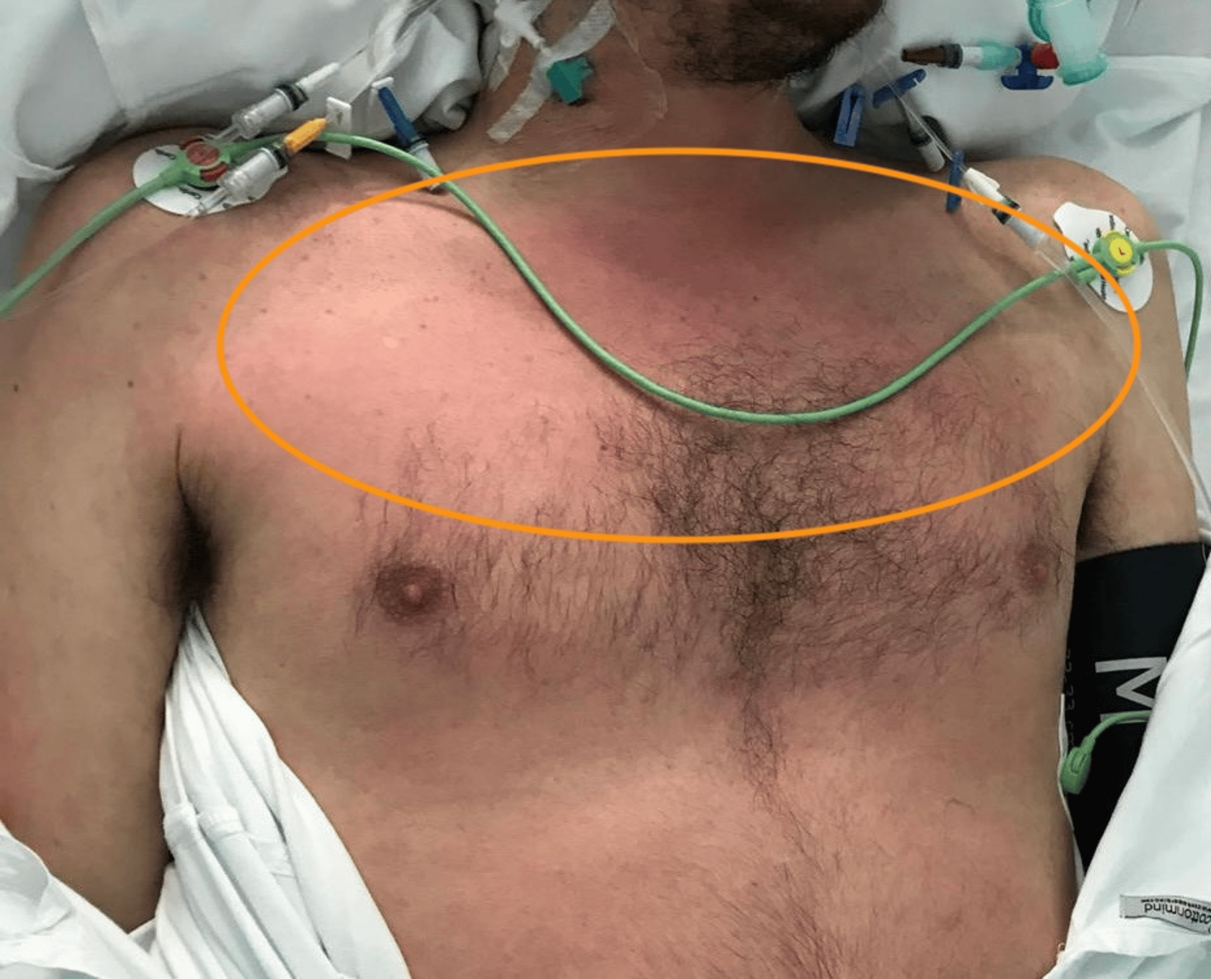
Coalescent maculopapular lesions on the trunk (orange circle).

**Figure 3:**
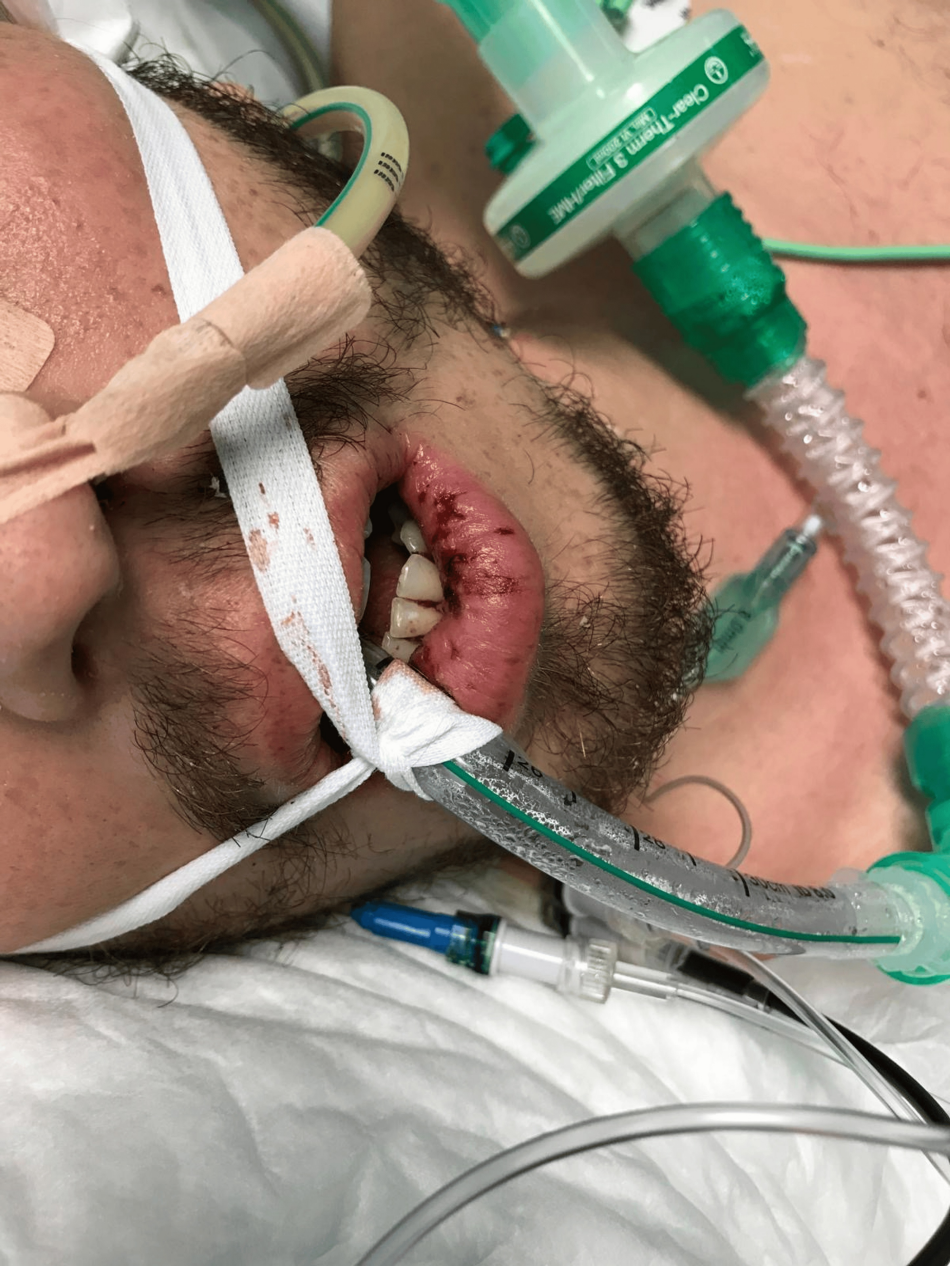
Mucositis of the lips, with hyperemia and fissures.

During hospitalization, a progression of the cutaneous rash was noticed, consisting of coalescent maculopapular lesions predominantly on the trunk (Figure 2), accompanied by persistent fever, diffuse conjunctival hyperemia, mucositis of the lips (Figure 3), and neck adenopathy. The patient remained dependent on vasopressor support despite three days of empiric antibiotics. The blood tests are described in Table 3. 

**Table 3:** Blood tests in intensive care unit INR: international normalized ratio

Laboratory test	Result	Normal range
Platelets	976000/uL	150000-400000/uL
Creatinine	0.64 mg/dl	0.7-1.3 mg/dl
Urea	49 mg/dl	15-39 mg/dl
INR	1.28	0.8-1.3 (may differ depending on the target)
Total bilirubin	0.64 mg/dl	0.2-1.0 mg/dl

**Figure 4:**
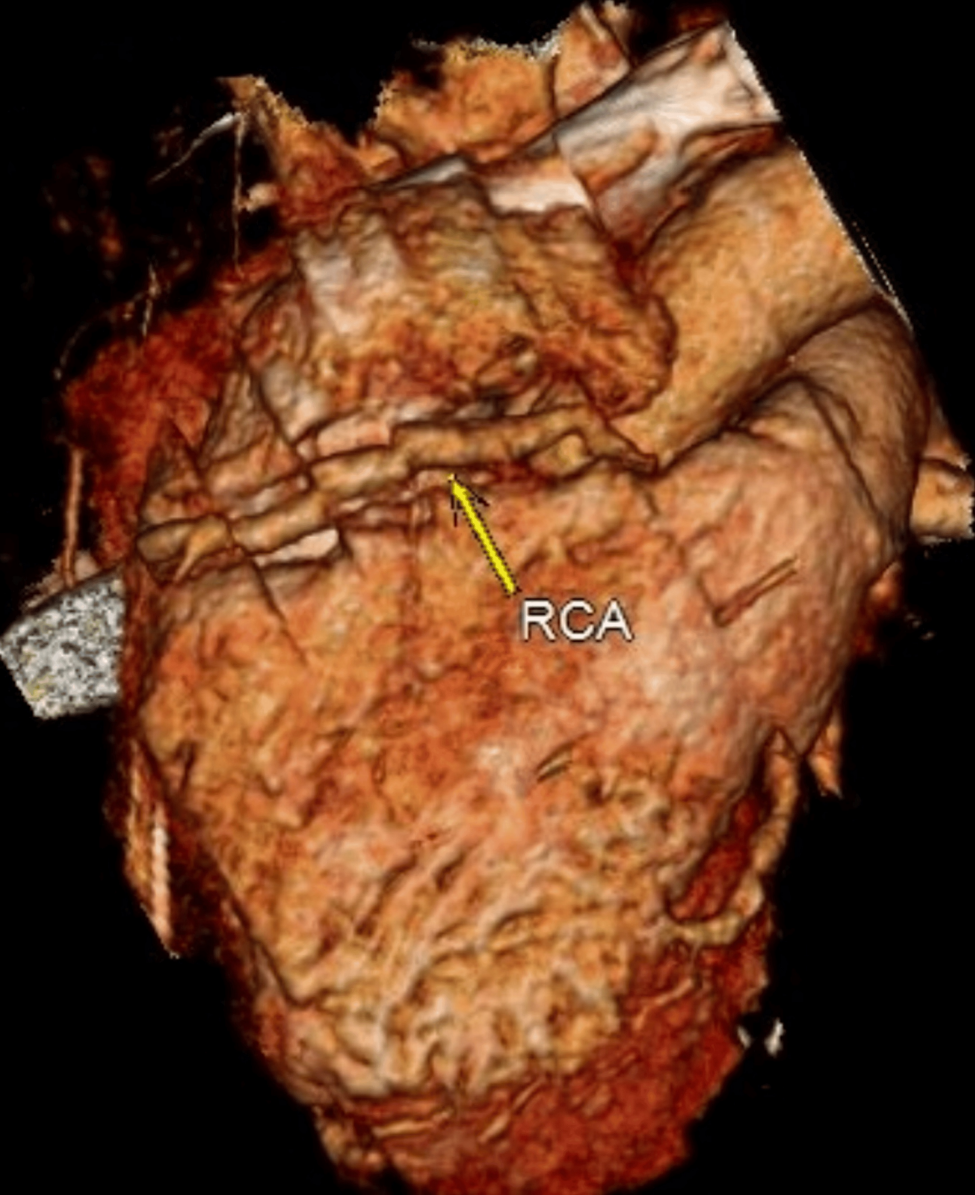
Coronary aneurisms affecting the right coronary artery (RCA) RCA: Aneurysmal dilation of the mid and distal segments, with a maximum diameter of 8x9 mm.

**Figure 5:**
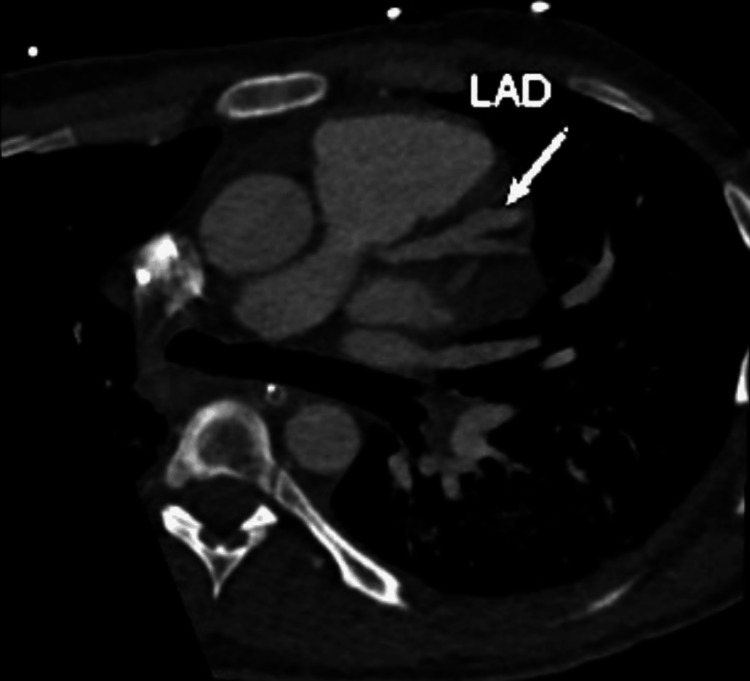
Coronary aneurisms affecting left anterior descending (LAD) artery LAD: marked dilation at the proximal/mid transition, with a maximum artery diameter of 9-10 mm.

A transthoracic echocardiogram showed inferior wall hypokinesia with preserved left ventricular ejection fraction, along with no valvular abnormalities. Considering this constellation of signs, a diagnosis of KD was considered, and he underwent coronary CT angiography (CTA), which showed multiple large (maximum 10 mm) coronary aneurysms affecting the left descending and right coronary arteries (Figure 4 and Figure 5). Thus, a diagnosis of adult-onset KD was assumed two weeks after the initial emergency room admission. 

The patient was started on a single dose of intravenous immunoglobulin (IVIg; 2g/kg) and corticosteroids (methylprednisolone 2mg/kg/day with weaning every five days) as well as acetylsalicylic acid (1g thrice a day). A marked symptomatic improvement was observed, leading to hemodynamic stability, vasopressor suspension, and fever resolution. After the diagnosis of KD, the patient remained in the ICU for five days. He was discharged home five days after leaving the ICU with optimal functional capacity. Unfortunately, the patient subsequently abandoned follow-up, precluding reassessment of the coronary lesions.

## Discussion

KD is an acute systemic vasculitis that primarily affects children, but its presentation in adults is rare and often challenging [1,3]. This case illustrates the diagnostic difficulties and complexity of management in an adult patient with atypical KD, highlighting the importance of early recognition to prevent severe complications.

Adult KD is less well understood and may present insidiously, particularly when compared to pediatric cases [4]. This was evidenced in our patient, who was initially diagnosed with pharyngitis, and 14 days were required to achieve a final KD diagnosis. Another conundrum lies in the fact that the classic diagnostic criteria, including persistent fever and clinical manifestations, such as conjunctivitis, mucositis, and cutaneous rash, may not present simultaneously in adults [1-6]. Furthermore, several clinical characteristics of KD, especially in serious cases, overlap with other entities, particularly toxic shock syndrome. Although the absence of a typical streptococcal or staphylococcal infection is suggestive, the presence of tonsillitis as a KD manifestation is often confounded with bacterial tonsillitis, which, albeit rarely, may cause toxic shock syndrome. The delay in the diagnosis of KD can lead to treatment delays and increase the risk of cardiovascular complications, such as coronary aneurysms. Indeed, the lack of familiarity among clinicians regarding the possibility of adult-onset KD may be a primary cause of underdiagnosis [5,6]. 

Our patient presented with shock, necessitating an aggressive approach, including mechanical ventilation and hemodynamic support [4]. However, the development of other multisystemic findings (e.g., conjunctivitis, mucositis, and lymphadenopathy), coupled with echocardiographic and coronary angiography results, ultimately allowed for the correct diagnosis of KD. The identification of coronary aneurysms, especially in a young patient, underscores the need for vigilance and consideration of KD in adults with suggestive clinical presentations, as up to 25% of patients with untreated KD and 5% of those treated with IVIg will develop coronary artery aneurysms [5,11].

Appropriate treatment, which included IVIg and glucocorticoids, resulted in favorable clinical response and hemodynamic stabilization. Current guidelines recommend that IVIg therapy be administered as early as possible to reduce the risk of cardiovascular complications, accompanied by intravenous glucocorticoids and high-dose acetylsalicylic acid [2,3,5].

This case emphasizes the importance of a multidisciplinary approach and ongoing clinician education regarding adult KD. Choosing an immunosuppressive strategy in patients with KD is particularly challenging when the initial diagnosis includes an infectious process. The overlap in clinical presentations necessitates careful consideration, as immunosuppression could exacerbate the underlying infection. Thus, a thorough differential diagnosis is vital to ensure that treatment decisions do not compromise patient safety. Increased awareness may improve early recognition rates and, consequently, effective management, minimizing the risk of long-term sequelae [5,6]. Furthermore, it highlights the necessity for careful differential diagnosis in patients with persistent fever and inflammatory manifestations, considering KD, among other conditions.

## Conclusions

KD may lead to serious complications, including coronary artery disease, if left untreated. Treatment includes early control of acute inflammation and monitoring for aneurysmal complications. Early recognition and appropriate treatment are crucial in preventing severe complications, reinforcing the need for a proactive approach to identifying and managing KD in adults.
